# Durability of a Vesicular Stomatitis Virus-Based Marburg Virus Vaccine in Nonhuman Primates

**DOI:** 10.1371/journal.pone.0094355

**Published:** 2014-04-23

**Authors:** Chad E. Mire, Joan B. Geisbert, Krystle N. Agans, Benjamin A. Satterfield, Krista M. Versteeg, Elizabeth A. Fritz, Heinz Feldmann, Lisa E. Hensley, Thomas W. Geisbert

**Affiliations:** 1 Galveston National Laboratory, University of Texas Medical Branch, Galveston, Texas, United States of America; 2 Department of Microbiology and Immunology, University of Texas Medical Branch, Galveston, Texas, United States of America; 3 Laboratory of Virology, Division of Intramural Research, National Institute of Allergy and Infectious Diseases, National Institutes of Health, Hamilton, Montana, United States of America; 4 Integrated Research Facility at Fort Detrick, Division of Clinical Research, National Institute of Allergy and Infectious Diseases, National Institutes of Health, Frederick, Maryland, United States of America; Public Health Agency of Canada, Canada

## Abstract

The filoviruses, Marburg virus (MARV) and Ebola virus, causes severe hemorrhagic fever with high mortality in humans and nonhuman primates. A promising filovirus vaccine under development is based on a recombinant vesicular stomatitis virus (rVSV) that expresses individual filovirus glycoproteins (GPs) in place of the VSV glycoprotein (G). These vaccines have shown 100% efficacy against filovirus infection in nonhuman primates when challenge occurs 28–35 days after a single injection immunization. Here, we examined the ability of a rVSV MARV-GP vaccine to provide protection when challenge occurs more than a year after vaccination. Cynomolgus macaques were immunized with rVSV-MARV-GP and challenged with MARV approximately 14 months after vaccination. Immunization resulted in the vaccine cohort of six animals having anti-MARV GP IgG throughout the pre-challenge period. Following MARV challenge none of the vaccinated animals showed any signs of clinical disease or viremia and all were completely protected from MARV infection. Two unvaccinated control animals exhibited signs consistent with MARV infection and both succumbed. Importantly, these data are the first to show 100% protective efficacy against any high dose filovirus challenge beyond 8 weeks after final vaccination. These findings demonstrate the durability of VSV-based filovirus vaccines.

## Introduction

The family *Filoviridae* is comprised of two genera *Ebolavirus* (EBOV) and *Marburgvirus* (MARV) [1]. These viruses cause severe and often fatal hemorrhagic fever with case fatality rates ranging from 23–90% depending on the strain and/or species. MARV has been responsible for at least nine outbreaks since 1967 with six of these occurring in the last decade [2], including a recent outbreak which started in September 2012 in Uganda [3]. The increased frequency of MARV outbreaks together with the fact that this virus is a potential agent of bioterrorism has increased public health concern regarding filoviruses. Currently, there are no licensed vaccines or postexposure treatments available for human use. However, there are at least five different vaccine candidates that have shown the potential to protect nonhuman primates (NHPs) from lethal MARV infection when challenge occurs 28–42 days post-vaccination. These vaccines include DNA vectors, recombinant Adenovirus (rAd) vectors, combined DNA/rAd vectors, virus-like particles (VLPs), alphavirus replicons, and recombinant vesicular stomatitis virus (rVSV) [4], [5], [6].

The rVSV-MARV-GP vaccine platform, where the VSV glycoprotein (G) is replaced with the MARV GP, has shown efficacy as both a single-injection preventive vaccine [7], [8], [9], [10] and a postexposure treatment against MARV challenge in NHPs [11], [12]. Initial studies with these vaccines proved that a single intramuscular (i.m.) vaccination of cynomolgus macaques with the rVSV-MARV-GP vector induces a strong humoral immune response and elicits complete protection against a high dose (1000 plaque forming unit [PFU]) i.m. challenge with homologous MARV 28 days later [10]. This vaccine has also been shown to be 100% efficacious in NHPs against homologous aerosol challenge 28 days after vaccination [8]. We have also shown that a single injection of a blended vaccine consisting of equal parts of rVSV-*Zaire ebolavirus*-GP, rVSV-*Sudan ebolavirus*-GP, and rVSV-MARV-GP completely protected NHPs against lethal challenge with three different species of EBOV or MARV [9].

The durability of the immune response elicited by a vaccine is an important factor in determining the regimen used. For example, will a single injection or a booster series be needed to confer long lasting protection against the pathogen in question? To date there are at least 16 vaccines against human viruses that have been developed, shown to be efficacious, and approved for use in humans including adenovirus, hepatitis A virus, hepatitis B virus, human papillomavirus, influenza virus, Japanese encephalitis virus, measles virus, mumps virus, poliovirus, rabies virus, rotavirus, rubella virus, smallpox, tick-borne encephalitis virus, varicella-zoster virus, and yellow fever virus [13]. Each vaccine has been investigated for durability but not necessarily under similar test conditions. Of these vaccines durability has been examined by circulating immunoglobulin G (IgG) for hepatitis A virus [14], measles virus, [15], rabies virus [16], tick-borne encephalitis virus [17], varicella-zoster virus [18], rubella virus [19], and mumps virus [20].

Here, we evaluated the durability of the rVSV-MARV-GP vaccine to protect NHPs against MARV challenge more than one year after vaccination. In addition, the pre- and post-vaccination circulating IgG levels against MARV-GP were examined monthly throughout the study. To date this study represents the first evaluation of the durability of a vaccine against a highly pathogenic filovirus.

## Materials and Methods

### Ethics statement

Healthy, adult cynomolgus macaques (*Macaca fascicularis*) were handled in Animal BSL-2 and BSL-4 containment space in the Galveston National Laboratory (GNL) at the University of Texas Medical Branch (UTMB), Galveston, Texas. Research was conducted in compliance with the Animal Welfare Act and other federal statutes and regulations relating to animals and experiments involving animals, and adhered to principles stated in the Eighth edition of the Guide for the Care and Use of Laboratory Animals, National Research Council, 2013. The facility where this research was conducted (UTMB) is fully accredited by the Association for the Assessment and Accreditation of Laboratory Animal Care International and has an approved OLAW Assurance #A3314-01. Research was conducted under animal protocol numbers 1006028 and 1206037 approved by the UTMB Institutional Animal Care and Use Committee (IACUC). All steps were taken to ameliorate the welfare and to avoid the suffering of the animals in accordance with the “Weatherall report for the use of nonhuman primates” recommendations. Animals were housed in adjoining individual primate cages allowing social interactions, under controlled conditions of humidity, temperature, and light (12-hour light/12-hour dark cycles). Food and water were available ad libitum. Animals were monitored (pre- and post-infection) and fed commercial monkey chow, treats and fruit twice daily by trained personnel. Environmental enrichment consisted of commercial toys. All procedures were conducted by trained personnel under the oversight of an attending veterinarian and all invasive clinical procedures were performed while animals were anesthetized. Humane endpoint criteria was specified and approved by the UTMB IACUC. Specifically, we applied a scoring sheet that assessed clinical signs to determine the time of euthanasia. Clinical signs scored included respiratory distress, weakness, changes in behavior, and coagulation disorders. One of two control animals was euthanized when scoring criteria were met. This animal was euthanized under anesthesia using a pentobarbital-based euthanasia solution. All surviving animals were also euthanized at the study endpoint under anesthesia using a pentobarbital-based euthanasia solution.

### rVSV vaccine vector and challenge virus

**Figure 1:**
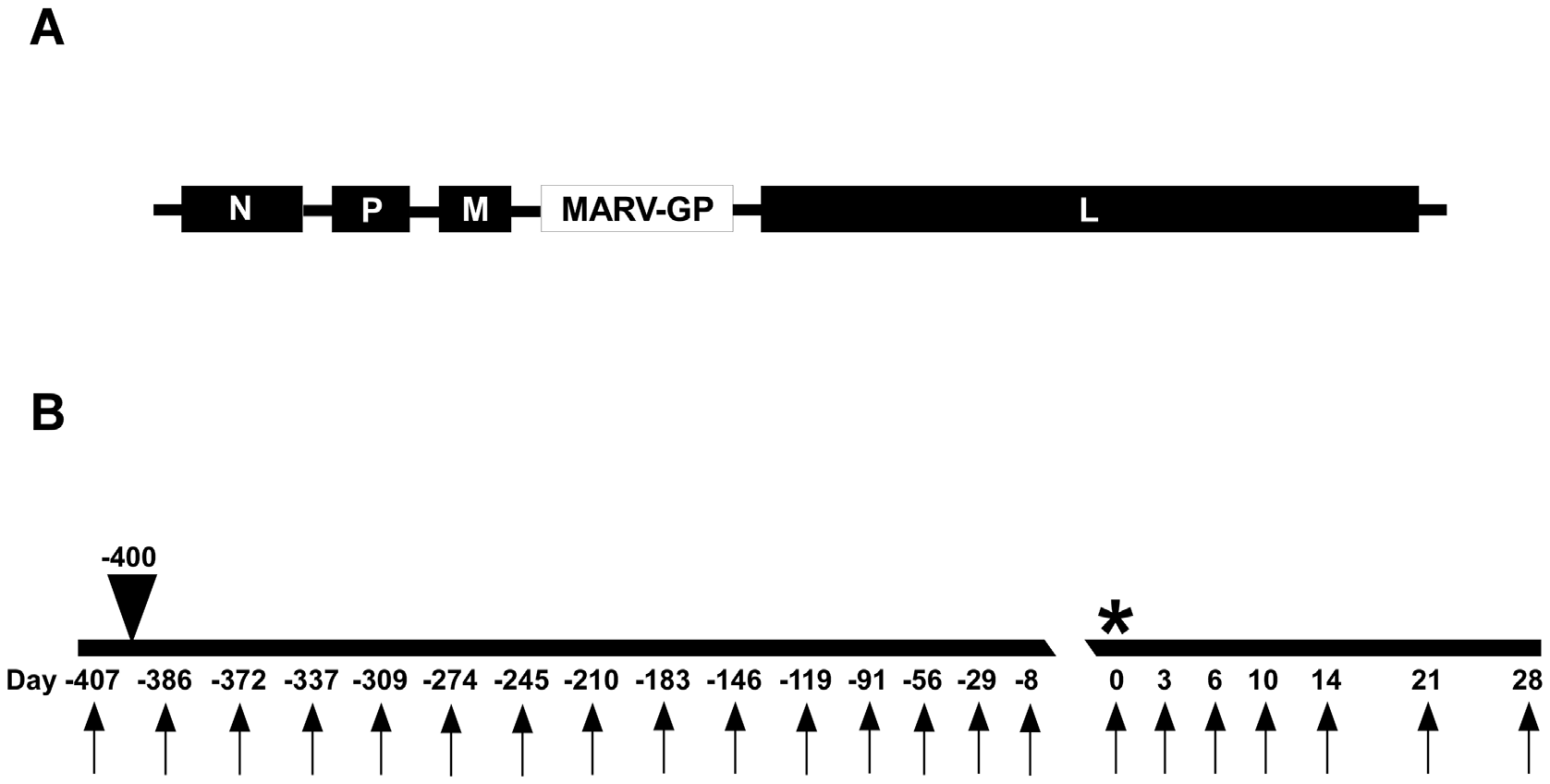
Vaccine and study design. (A) Diagram of the rVSV-MARV-GP genome used in the study design shown in B. (B) Diagram with sample days shown below (arrows), arrow head above depicting day of vaccination (-400), and * depicting the day of challenge (0).

The rVSV filovirus GP vector, rVSV-MARV-GP (Musoke strain), which has the MARV GP [Genbank accession no. NC_001608 Gene ID 920945] gene in place of the VSV glycoprotein (Fig. 1A), was recovered from cDNA as previously described [21], [22]. MARV-Musoke 803128, was isolated from a human case in Kenya in 1980 [23]. The challenge stock of MARV used in this study was propagated on Vero E6 cells four times making this a passage 4 virus. The MARV challenge stock was kindly provided by Dr. Thomas G. Ksiazek.

The rVSV-MARV-GP vector preparations and MARV challenge virus stocks were assessed for the presence of endotoxin using The Endosafe-Portable Test System (PTS) (Charles River, Wilmington, MA). Virus preparations were diluted 1∶10 in Limulus Amebocyte Lysate (LAL) Reagent Water (LRW) per manufacturer's directions and endotoxin levels were tested in LAL Endosafe-PTS cartridges as directed by the manufacturer. Each preparation was found to be below detectable limits while positive controls showed that the tests were valid.

### Immunization and challenge of nonhuman primates

Eight, healthy, filovirus-naïve, adult (5–10 years in age, 3–12 kg), male and female Chinese origin cynomolgus macaques (*Macaca fascicularis*) were randomly assigned to one group of six vaccinated animals and a control group of two non-vaccinated animals. Animals were individually caged in a BSL-4 laboratory animal room. Animals in the vaccine groups were vaccinated by i.m. injection with ∼2×10^7^ plaque-forming units (PFU) of rVSV-MARV-GP. Approximately fourteen months post-vaccination all animals were challenged i.m. using a historically lethal dose of 1,000 PFU of MARV (Musoke strain).

Animals were monitored for clinical signs of illness (temperature, weight loss, changes in blood count, and blood chemistries) during the vaccination and MARV challenge portions of the study. MARV viremia was analyzed after challenge. Physical exams were given when blood was collected on day -407, day -400, days -386 to -8 (monthly), and on days 0, 3, 6, 10, 14, 21, and 28 post-challenge (Fig. 1B, arrows).

### Hematology and serum biochemistry analysis

Total white blood cell counts, white blood cell differentials, red blood cell counts, platelet counts, hematocrit values, total hemoglobin concentrations, mean cell volumes, mean corpuscular volumes, and mean corpuscular hemoglobin concentrations were analyzed from blood collected in tubes containing EDTA using a laser based hematologic analyzer (Beckman Coulter, Brea, CA). Serum samples were tested for concentrations of albumin, amylase, alanine aminotransferase (ALT) aspartate aminotransferase (AST), alkaline phosphatase (ALP), gamma-glutamyltransferase (GGT), glucose, cholesterol, total protein, total bilirubin (TBIL), blood urea nitrogen (BUN), creatine (CRE), and C-reactive protein (CRP) by using a Piccolo point-of-care analyzer and Biochemistry Panel Plus analyzer discs (Abaxis, Sunnyvale, CA).

### Detection of viremia

RNA was isolated from whole blood utilizing the Viral RNA mini-kit (Qiagen, Valencia, CA) using 100 µl of blood into 600 µl of buffer AVL. Primers/probe targeting the L gene of MARV were used for quantitative real-time PCR (qRT-PCR) as used previously [7] with the probe used here being 6-carboxyfluorescein (6FAM)-5′CGCGGCATTTCA3′-6 carboxytetramethylrhodamine (TAMRA) (Life Technologies, Grand Island, NY). MARV RNA was detected using the CFX96 detection system (BioRad Laboratories, Hercules, CA) in One-step probe qRT-PCR kits (Qiagen) with the following cycle conditions: 50°C for 10 minutes (min), 95°C for 10 seconds (s), and 40 cycles of 95°C for 10 s and 59°C for 30 s. Threshold cycle (*CT*) values representing MARV genomes were analyzed with CFX Manager Software, and data are shown as + or − for genome equivalents (GEq) (Table 1). To create the GEq standard, RNA from MARV stocks was extracted and the number of MARV genomes was calculated using Avogadro's number and the molecular weight of the MARV genome.

**Table 1:** Reciprocal MARV GP serum neutralizing antibody titers at which 50% of rVSV-MARV-GP was neutralized.

Animal	Vaccine	Day -407^a^	Day -372^a^^,^ b	Day -210^a^	Day 0^a^	Terminal^c^
						
**C050960** *	None	n.d.	n.d.	n.d.	≤10	≤10
						
**C061226** *	None	n.d.	n.d.	n.d.	≤10	≤10
						
**10–230**	rVSV-MARV-GP	≤10	**40**	≤10	**40**	**80**
						
**119–177**	rVSV-MARV-GP	≤10	**80**	**20**	**20**	**80**
						
**0406033**	rVSV-MARV-GP	≤10	**40**	≤10	**40**	**40**
						
**0401043**	rVSV-MARV-GP	≤10	**40**	≤10	≤10	**40**
						
**0410077**	rVSV-MARV-GP	≤10	**20**	≤10	≤10	**160**
						
**0212131**	rVSV-MARV-GP	≤10	**40**	**80**	**40**	**20**
						

*; Succumbed to MARV challenge.

n.d.; No data.

a Days after MARV challenge.

b Day 28 post rVSV-MARV-GP vaccination.

c See Table 1 for Terminal sample day of animals with a *; all others are from Day 28.

Virus titration was performed by plaque assay with Vero E6 cells from all serum samples as previously described [10]. Briefly, increasing 10-fold dilutions of the samples were adsorbed to Vero E6 monolayers in duplicate wells (200 µl); the limit of detection was 25 PFU/ml.

### Humoral immune response to MARV GP

Serum samples collected at indicated time points were tested for IgG antibodies against MARV GP. Enzyme-linked immunosorbent assay (ELISA) using a recombinant MARV GP delta transmembrane (dTM) purified protein (Integrated BioTherapeutics Inc., Gaithersburg, MD) was used to detect cross-reactive IgG. The MARV GPdTM was diluted to an optimal working concentration of 0.08 µg/mL and was coated on 96 well ELISA plates. Plates were blocked for non-specific binding 2 hours before the serum samples were assayed at 2-fold dilutions starting at a 1∶100 dilution in ELISA diluent (1% heat inactivated fetal bovine serum (HI-FBS), 1× PBS, and 0.2% Tween-20) in triplicate. Samples were incubated for 1 hour at room temperature (RT), removed, and plates were washed. Wells were then incubated at RT for 1 hour with anti-monkey IgG conjugated to horseradish peroxidase (Fitzgerald Industries International, Acton, MA) at a 1∶2500 dilution. These wells were washed and then incubated with 2,2′-azine-di(3ethylbenzthiazoline-6-sulfonate) peroxidase substrate system (KPL, Gaithersburg, MD) and read for dilution endpoints at 405 nm on a Molecular Devices Emax system microplate reader (Molecular Devices, Sunnyvale, CA). Antibody titers represented were defined as the highest reciprocal dilution with an optical density ≥0.2.

Neutralizing antibody titers were determined by performing plaque reduction neutralization titration assays (PRNT). Briefly, Vero cells were seeded into 6 well plates to generate a confluent monolayer on the day of infection. Serum dilutions were prepared in DMEM and 100 µL were incubated with ∼100 pfu of rVSV-MARV-GP in a total volume of 200 µL. Media was removed from cells, the serum-virus mixture was added, and samples were incubated for 60 min at 37°C. The mixture was removed from the cells and 2 ml of 0.9% agarose in EMEM with 5% FBS was overlayed onto the wells. Cells were observed 72 hours post-incubation and plaques were counted. The neutralizing antibody titer of a serum sample was considered positive at a dilution showing a ≥50% reduction (PRNT_50_) compared with the virus control without serum.

### Cellular immune response to MARV GP

T-cell responses were measured as previously shown for MARV [10] at days 14 and 28 after vaccination. Briefly, peripheral blood mononuclear cells were isolated from cynomolgus monkey whole blood samples by separation over Ficoll (Sigma-Aldrich, St. Louis, MO). Approximately 1×10^6^ cells were stimulated in 200 µl RPMI medium (Life Technologies) for 6 h at 37°C with anti-CD28 and anti-CD49d antibodies and either DMSO or a pool of 15-nucleotide peptides spanning the MARV GP (Musoke strain) open reading frames (Integrated DNA Technologies, Coralville, IA) in the presence of brefeldin A. The peptides were 15 amino acids in length, overlapping by 11 and spanning the entire MARV GP at a final concentration of 2 µg/ml. Cells were fixed and permeabilized with FACS lyse (Becton Dickinson, Franklin Lakes, NJ) containing Tween-20, and stained with a pool of antibodies against lineage markers (CD3-PE, CD4-PerCP, CD8-FITC) and either TNF-α-APC or IFN-γ-APC. Samples were run on a FACSCanto (Becton Dickinson) and analyzed using the software FlowJo (Tree Star, Inc., Ashland, OR). Positive gating for lymphocytes using forward versus side scatter was followed by CD3+/CD8− and CD3+/CD4− gating, and specific populations were further defined by anti-CD4 and anti-CD8 positivity, respectively. Cytokine-positive cells were defined as a percentage within these individual lymphocyte subsets, and at least 200,000 events were analyzed for each sample.

### Statistics

Survival was compared between vaccinated and non-vaccinated animals using a one tailed Fisher Exact Test. The 6 vaccinated animals were compared with the 2 control animals. For ethical considerations, the 2 controls were supplemented with 3 historical controls subjected to the same test conditions so that the control group consisted of 5 animals for statistical analysis.

## Results

### Humoral and cellular immune responses

**Figure 2:**
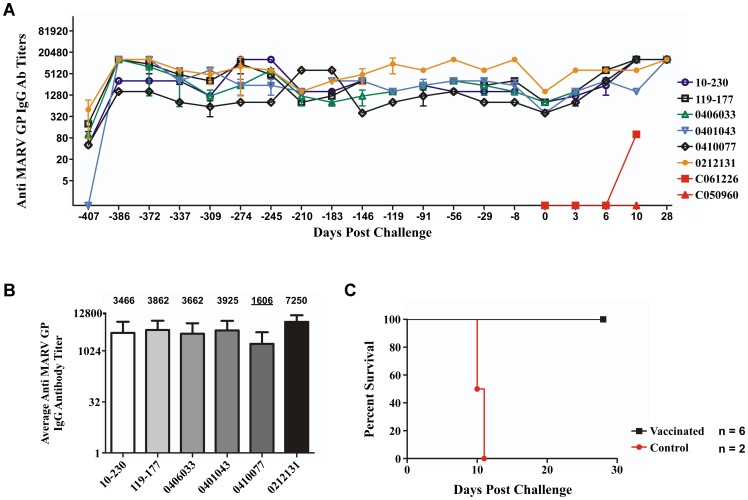
Circulating anti-MARV GP IgG and survival of vaccinated and control groups. (A) An ELISA was performed to measure the mean reciprocal titer of circulating anti-MARV GP IgG for each individual in the vaccinated cohort over the course of the 13 months before MARV challenge -407 to -8 and circulating anti-MARV GP IgG on the day of challenge (0) and days post-challenge for the non-vaccinated controls (red) and vaccinated cohort (top 6 in legend). Error bars represent standard deviation of samples in triplicate. (B) The average, over 13 months post-vaccination, mean reciprocal titer of circulating anti-MARV GP IgG before MARV challenge for each individual in the vaccinated cohort. Numerical value of average mean reciprocal titer displayed above individual bars in graph. Lowest average titer underlined. (C) Kapplan-Meier survival curve for the non-vaccinated control (red, n = 2)) and vaccinated groups (black, n = 6).

No adverse events were associated with the vaccination phase of the study. While previous studies of cynomolgus monkeys vaccinated with the rVSV-MARV-GP have failed to detect a cellular immune response elicited by vaccination [10], a strong humoral response has been associated with this vaccine [10], [24]. To evaluate the magnitude and longevity of anti-MARV GP IgG in NHPs vaccinated with rVSV-MARV-GP, we sampled the six vaccinated animals seven days before vaccination (Fig. 1B, Day -407) and on multiple days post-vaccination as shown in Figure 1B, arrows. At day -386 (day 14 post-vaccination) and from thereafter monthly, serum samples were examined for circulating anti-MARV GP IgG using an ELISA based on the MARV GPdTM. As expected, an increase in mean reciprocal IgG titers against MARV GP was observed at days -386 and -372 (days 14 and 28 post-vaccination respectively) as seen previously [7] with animal 0410077 having a titer at 1600, animal 110–230 at 3200, and the remaining animals in the vaccinated cohort at 12800 (Fig. 2A). The serum that was examined throughout the course of the year post-vaccination exhibited circulating anti-MARV GP IgG in every vaccinated animal with each oscillating between the lower and higher levels by mean reciprocal dilution (Fig. 2A, -407 to -8). While there was some oscillation of circulating anti-MARV GP IgG over the course of the year before challenge, the overall average antibody titer ranged from 1606 to 7250 as seen in Figure 2B.

The circulating antibodies were further characterized for their neutralizing activity with the vaccinated cohort of animals showing no neutralizing antibody titer before vaccination (Table 1, Day -407) and low PRNT_50_ titers ranging from 1∶20 to 1∶80 at day 28 post-vaccination (Table 1, Day -372). Just as the circulating anti-MARV GP IgG titer fluctuated over the course of the year, slight neutralizing antibody titer differences were observed between 7 months post-vaccination (Table 2, Day -210) and day of MARV challenge (Table 1, Day 0). In fact, two of the six animals had no detectable neutralizing antibody titers on the day of MARV challenge.

**Table 2:** Clinical findings and viremia for NHPs challenged with MARV.

**Animal**	**Vaccine**	**Symptoms Observed Between Day 0 and 28 after MARV challenge^a^**	**Clinical Score^c^**	**Serum Viremia^d^**	**Final Outcome**
**C050960**	None	Moderate rash (9–11), Anorexia (7–11), Depression (7–11) Mild rectorrhagia (10), Lymphopenia (6), ALP→→ → (10), AST→(6), AST→→→ (10), BUN→ (10), GGT→→ (10)	**1** (7), **1** (8), **1** (9), **4** (10), **15** (11)	**6.1**/+++ (6) **6.8**/+++ (10)	Died on Day 11
**C061226**	None	Moderate rash (8–10), Anorexia (7–10), Depression (7–10), Rectorrhagia (10), Lymphopenia (6), ALP→→→ (10), AST→→ (6), AST→→→ (10), BUN→ (10), GGT→→ (10)	**1** (7), **1** (8), **3** (9), **10** (10)	**6.9**/+++ (6) **7.3**/+++ (10)	Died on Day 10
**10–230**	rVSV-MARV-GP	Ø^b^	Ø	0/−	Survived
**119–177**	rVSV-MARV-GP	Ø	Ø	0/−	Survived
**0406033**	rVSV-MARV-GP	Ø	Ø	0/−	Survived
**0401043**	rVSV-MARV-GP	Ø	Ø	0/−	Survived
**0410077**	rVSV-MARV-GP	Ø	Ø	0/−	Survived
**0212131**	rVSV-MARV-GP	Ø	Ø	0/−	Survived

a Days after MARV challenge are in parentheses. Fever is defined as a temperature more than 2.5^O^F over baseline or at least 1.5^O^F over baseline and ≥103.5^O^F. Moderate rash refers to petechiae coverage of more than 20% of the skin. Lymphopenia and thrombocytopenia are defined by a ≥35% drop in numbers of lymphocytes and platelets, respectively. (ALP) alkaline phosphatase, (AST) aspartate aminotransferase, (BUN) blood urea nitrogen, (GGT) gamma glutamyltransferase: 2- to 3-fold increase,→; 4- to 5-fold increase, →→; >5 fold increase, →→→.

b No symptoms observed.

c Clinical scores in bold type with day of score in parentheses. Clinical scores were recorded each day post-challenge for each animal using a scoring system based on dyspnea, depression, recumbency, and rash. A clinical score ≥9 was the criteria for euthanasia per IACUC protocol.

d Days after MARV challenge are in parentheses. Viral load for each MARV positive day is depicted as: log_10_ PFU/ml**/**qRT-PCR positive (+) or negative (−). +, ≤5 log_10_; ++, ≥6 log_10_; +++, ≥7 log_10._

### MARV challenge

The mortality rate for the cynomolgus macaque model after MARV (Musoke strain) challenge is 100%. To test whether durable immunity could be induced against MARV challenge over one year after vaccinating with the rVSV-MARV-GP vaccine, we challenged the vaccinated group and non-vaccinated group of cynomolgus monkeys with a lethal dose of MARV 14 months post-vaccination (Fig. 1B, *). The animals were closely monitored over the course of 28 days post-challenge for clinical signs of illness. The vaccinated cohort of six animals was 100% protected (6/6) against MARV (Fig. 2C, black), while the two animals in the non-vaccinated control group succumbed to infection on days 10 and 11, respectively (Fig. 2C, red, Table 2). Protection was statistically significant (P = 0.0022). Clinical scores were recorded each day post-challenge for each animal using a scoring system based on dyspnea, depression, recumbency, and macular rash. The clinical scores for each animal correlated with the survival data as seen with the mean clinical score for each animal in the vaccinated group having no score on any day post-challenge and the non-vaccinated group having clinical scores on days 7 to 11 post-challenge (Table 2).

The non-vaccinated control animals exhibited typical sequelae in response to MARV infection such as macular rash, anorexia, depression, fever, increase in liver enzymes, and thrombocytopenia, whereas the vaccinated cohort showed no signs of disease (Table 2). This observation correlates well with the fact that infectious MARV and MARV RNA was only isolated from sera of the two animals in the non-vaccinated group on days 6 and 10 post-challenge (Table 2).

The levels of anti-MARV GP circulating IgG antibody were also evaluated in the sera of the control and vaccinated animals at sampling days post-challenge (Fig. 2A, 0 to 28). The control animals had no appreciable mean reciprocal anti-MARV GP IgG titers on any day post-challenge (Fig. 2A, red), whereas the vaccinated group had IgG titers which increased from day 0 to day 28 post-challenge for all animals (Fig. 2A, 0 to 28). As seen with the circulating anti-MARV GP IgG, the vaccinated cohort of animals showed an increase in neutralizing antibody titers at day 28 post-challenge revealing a productive immune response to MARV challenge (Table 1, Terminal).

## Discussion

Almost a decade ago, rVSV vectors expressing foreign GPs from EBOV and MARV were developed and characterized [22]. These rVSV vaccine vectors have since been used in cynomolgus and rhesus macaques and shown to be highly efficacious against a lethal challenge with three different species of EBOV and MARV [7], [8], [9], [10], [25], [26]. To date, the rVSV filovirus GP vectors have been used in over 100 NHPs with no signs of toxicity as a result of vaccination [7], [8], [9], [10], [24], [25], [26], [27]. In addition, the rVSV-ZEBOV-GP vector was recently used as a treatment less than 48 hours after a possible, accidental ZEBOV exposure to a laboratory worker in Germany. While the efficacy of the treatment was not conclusive, the treated individual experienced mild fever, myalgia, and headache 12 hours after injection [28]. Although these data suggested that the vectors were innocuous, the use of the rVSV-filovirus-GP vectors as vaccines required further safety testing; the rVSV-ZEBOV-GP vaccine was well tolerated in a SHIV macaque model [25] and more recently we subjected the rVSV-ZEBOV-GP and rVSV-MARV-GP vaccines to a neurovirulence test in NHPs where the vectors displayed no neurovirulence when compared to wild type rVSV [27].

While the efficacy and safety of the rVSV-filovirus-GP vaccines have been studied, the ability of these vaccines to protect against challenge at times longer than 4 weeks after vaccination has yet to be explored. Here, we assessed the ability of the rVSV-MARV-GP vaccine to protect cynomolgus macaques, the "gold-standard" filovirus vaccine model, from MARV challenge over one year after vaccination. To our knowledge, this is the first study to investigate whether a vaccine against a filovirus can protect NHPs, challenged with a filovirus, over one year after vaccination. The vaccines were indeed able to protect a cohort of NHPs challenged with MARV beyond one year post-vaccination with rVSV-MARV-GP. No evidence of a cellular immune response was detected at days 14 or 28 after vaccination (data not shown). This result is consistent with our previous studies with VSV-based MARV vaccines [10] and provides further support that protection in the cynomolgus monkey model is not solely dependent on cellular immunity or assays used to date are not sensitive enough to detect a response. However, it is important to note that we were able to detect circulating anti-MARV GP IgG over one year after rVSV-MARV-GP vaccination from a single injection which is encouraging considering that the ability to vaccinate an "at risk" population in an endemic area at least one time, would be more feasible than a multiple dose regimen. Over the course of the year after vaccination, all animals reached peak IgG titers of 12800 with the exception of animal 0410077 which reached a peak titer of 6400 before challenge. However, all animals in the vaccinated cohort reached a titer of 12800 by 28 post-challenge. Previous studies using the rVSV-MARV-GP vector, which completely protected 15 cynomolgus macaques from MARV challenge [7], [8], [10], had IgG titers which ranged from 100 to 1000 with an average of 760. The titers measured in this study were measured against soluble MARV-GP versus inactivated virus particles as done previously [7], [8], [10] which may account for the higher titers measured herein as the plates were most likely coated with more antigen to detect anti-MARV GP IgG. IgG titers to ZEBOV-GP after vaccinations against the antigen which correlate with 100% survival against ZEBOV challenge have been measured to at least 3700 [29] and while all animals in the vaccinated cohort during this study reached levels above 3700 the average titer of 1606 for animal 0410077 was below this level though it peaked at 6400 before challenge. From these data it appears that production of anti-MARV-GP IgG correlates with protection against MARV challenge including an average titer of 1606 which is below the correlate for ZEBOV.

Our data are also encouraging, considering a recent study uncovered the mechanism of protection for the rVSV-ZEBOV-GP vaccine in a lethal ZEBOV NHP model [24]. It was shown that antibodies were necessary for protection against ZEBOV challenge using the rVSV-filovirus-GP platform [24]. Here, we report on the circulation of anti-MARV-GP IgG and low levels of neutralizing antibodies against MARV GP (Table 2). One can hypothesize that protection against MARV challenge 14 months post-vaccination correlates with the circulating anti-MARV-GP antibody levels with the role of neutralizing antibodies being minimal to non-existent. Antibody-dependent cell-mediated cytotoxicity (ADCC) and antibody-dependent complement-mediated cytotoxicity (ADC′C) are mechanisms by which antibodies can act as protective non-neutralizing monoclonal antibodies (PnNMAbs) by destroying cells with antigen bound by antigen specific antibodies [30]. The disproportion of antibody levels to neutralizing antibodies for filovirus protection suggests these mechanisms may be prominent in vaccine protection against challenge as reported previously for virus-like particle vaccines in NHPs [31] and may be part of the mechanism for rVSV-filovirus-GP vaccine protection.

We initially posited that the use of the rVSV-MARV-GP vaccine would be in the manner of a ring vaccination strategy in response to a MARV outbreak where the population surrounding the epicenter would be immunized to MARV to contain the spread of the virus; much like the strategy used to contain smallpox outbreaks. The typical immunization period for this vaccine in NHPs is 28 days although recently it has been reduced to 21 days (TWG, unpublished data). This would mean that the population surrounding the epicenter or any other concerned population could potentially be protected within 3 weeks of a confirmed outbreak. While the results of this study have not changed the feasibility of the rVSV-MARV-GP vaccine to be used in a ring vaccination strategy, we have shown that vaccination with the vaccine produced a productive immune response which afforded protection against MARV beyond one year post-vaccination. While we are encouraged by the durability of the rVSV-MARV GP vaccine against MARV-Musoke challenge the durability against the seemingly more pathogenic Angola strain of MARV still needs to be addressed [32], [33]. The durability of the rVSV-ZEBOV-GP and rVSV-SEBOV GP vaccines should also be considered in the future but with these initial data we are encouraged about the potential for protection against filoviruses one year post-vaccination using the rVSV-filovirus-GP platform.

## Supporting Information

Checklist S1 ARRIVE Guidelines Checklist.
(DOC)
